# Ultra rapid *in vivo* screening for anti-Alzheimer anti-amyloid drugs

**DOI:** 10.1038/srep23349

**Published:** 2016-03-22

**Authors:** Alba Espargaró, Aina Medina, Ornella Di Pietro, Diego Muñoz-Torrero, Raimon Sabate

**Affiliations:** 1 Department of Physical Chemistry, School of Pharmacy, and Institute of Nanoscience and Nanotechnology (IN2UB), University of Barcelona, Spain; 2 Laboratory of Pharmaceutical Chemistry (CSIC Associated Unit), School of Pharmacy, and Institute of Biomedicine (IBUB), University of Barcelona, Spain

## Abstract

More than 46 million people worldwide suffer from Alzheimer’s disease. A large number of potential treatments have been proposed; among these, the inhibition of the aggregation of amyloid β-peptide (Aβ), considered one of the main culprits in Alzheimer’s disease. Limitations in monitoring the aggregation of Aβ in cells and tissues restrict the screening of anti-amyloid drugs to *in vitro* studies in most cases. We have developed a simple but powerful method to track Aβ aggregation *in vivo* in real-time, using bacteria as *in vivo* amyloid reservoir. We use the specific amyloid dye Thioflavin-S (Th-S) to stain bacterial inclusion bodies (IBs), in this case mainly formed of Aβ in amyloid conformation. Th-S binding to amyloids leads to an increment of fluorescence that can be monitored. The quantification of the Th-S fluorescence along the time allows tracking Aβ aggregation and the effect of potential anti-aggregating agents.

Amyloid aggregation is linked to an increasing number of human diseases, including both non-neurologic and neurodegenerative disorders^1^. These human disorders, grouped under the term “conformational diseases”, include Alzheimer’s (AD), Parkinson’s (PD) and Huntington’s (HD) diseases, frontotemporal dementia (FTD), amyotrophic lateral sclerosis (ALS) or type II diabetes, among others^1^. Nowadays, more than 46 million people worldwide suffer from AD and the number is predicted to exceed 130 million by 2050^2^,^3^. AD is a multifactorial and highly complex process, whose pathogenesis involves multiple mechanisms^4^. However, the appearance of both, amyloid plaques –consequence of the accumulation of amyloid β-peptide (Aβ)– and neurofibrillary tangles –mainly formed of hyperphosphorylated forms of tau protein from neuronal microtubules– are the most prominent pathological hallmarks in the brain of AD patients, leading to neuronal cell death and tissue loss throughout the brain^5^. For years it has been discussed which is the main cause of Alzheimer’s disease. Currently Aβ aggregation is widely accepted to be one of the main culprits of the illness^6^,^7^. In this light, in the last few years the search for potential inhibitors of amyloid aggregation has become one of the most pursued therapeutic strategies in the fight against AD^8^,^9^,^10^,^11^.

A large number of methods to track the amyloid aggregation have been recently proposed^12^,^13^,^14^,^15^,^16^. The evaluation of potential anti-amyloid drugs is usually hampered by the lack of physiologically relevant methods that can be easily implemented in high-throughput screening. Monitoring of amyloid aggregation in cells and tissues suffers from important drawbacks arising from low protein concentration, slow aggregation process and low reproducibility^17^. These limitations have restricted the screening of anti-amyloid compounds to *in vitro* studies using expensive synthetic peptides^17^. Currently, only a handful of methods, which usually track the amyloid associated toxicity in cell lines or the amyloid aggregation *in vitro*, are used to screen anti-Alzheimer anti-amyloid drugs^18^,^19^,^20^,^21^. However, the *in vitro* Aβ aggregation is far from *in vivo* conditions. Moreover, the cellular toxicity is not directly related to the final amyloid amount, but to the type of amyloid-like aggregates formed during the aggregation process. In fact, soluble Aβ oligomers, generated at the early stages of the fibrillation process, are considered the primary cytotoxic species^1^,^22^,^23^,^24^. Because *in vivo* amyloid kinetics may provide key information about both the amyloid aggregation process, still essentially uncharacterized, and inhibition mechanisms, the development of fast, simple, reproducible *in vivo* methods could represent a breakthrough in the understanding of the amyloid aggregation process and eventually in the search for potential anti-Alzheimer anti-amyloid drugs. Bacteria represent a simple but quantitative method, which will always be closer to the *in vivo* conditions in mammals than *in vitro* and toxicity assays.

The proposed method uses bacteria as an *in vivo* reservoir to track in real-time amyloid aggregation kinetics. The use of prokaryotic systems as microbial cell factories in the production of recombinant proteins has become an essential tool for the biotechnological industry and biomedical research^25^. Over-expression of amyloid-prone proteins in bacteria entails the formation of insoluble protein aggregates called inclusion bodies (IBs)^26^,^27^. Compelling evidence has unequivocally demonstrated that recombinant amyloid-prone proteins are folded in amyloid-like conformations into IBs^26^,^27^. Indeed, IBs formed after the over-expression in bacteria of the major Aβ peptides, namely Aβ40 and Aβ42, have been shown to display amyloid-like structures^28^. In this context, we have recently shown the usefulness of the amyloid specific dye Thioflavin-S (Th-S) to track the amyloid deposits of different amyloid-prone proteins in bacteria^29^,^30^. The facts that (1) Th-S staining of amyloid-like structures leads to an increase in it specific fluorescence –when excited under blue light– which can be easily monitored without interfering the bacteria growth and (2) Th-S crosses membranes and penetrates into the cell without affecting amyloid aggregation, make Th-S the dye of choice for tracking amyloid aggregation *in vivo*^29^,^30^. Despite the noticeable applicability of our previously reported methods in anti-amyloid drug discovery, they do not allow the quantification of the amyloid amount in cell or the amazing possibility to track in-real time the *in vivo* aggregation of amyloid-prone proteins. Herein, we describe an alternative method that compiles the capacity of bacteria as physiologically relevant models for the *in vivo* study of amyloid aggregation and the ability of Th-S to stain amyloid structures, thereby enabling the quantitative *in vivo* screening of anti-amyloid drugs, both in kinetic and thermodynamic terms. Importantly, the proposed method allows to track simply but effectively the inhibitory capacity of potential anti-amyloid drugs at each stage of the aggregation process, providing direct information of the behavior of each inhibitor along the aggregation time.

## Results and Discussion

**Figure 1:**
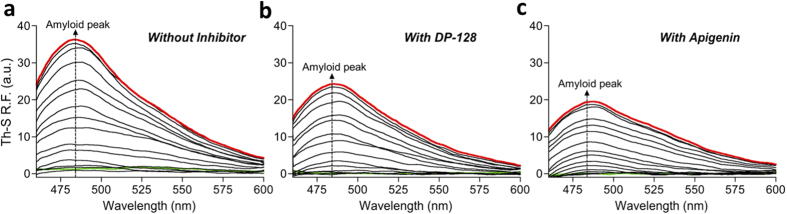
Th-S relative fluorescence of the amyloid band along the time-course. (**a**) In the absence of inhibitor. (**b**) In the presence of 10 μM DP-128. (**c**) In the presence of 10 μM apigenin. The dotted arrows show the maximal amyloid peak ~485 nm. In green and red, the initial (0 min) and final (480 min) time-course. Th-S relative fluorescence measurements were performed in triplicate and the standard errors were less than 5%.

**Figure 2:**
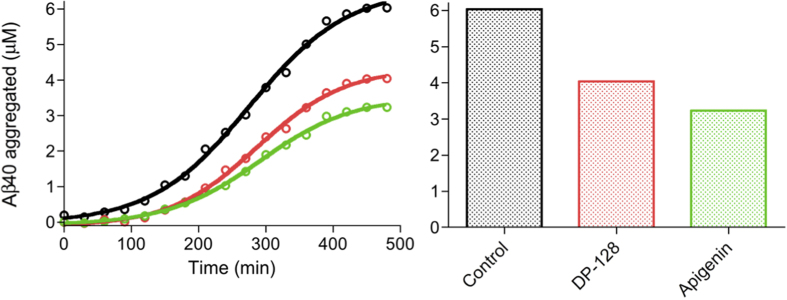
Aβ40 amyloid concentration along the time-course kinetics and amyloid concentration at end-point of the time-course. In black, red and green, in the absence (control) and presence of 10 μM DP-128 and apigenin, respectively. Aβ40 concentrations were measured in triplicate and the standard errors were less than 5%.

**Figure 3:**
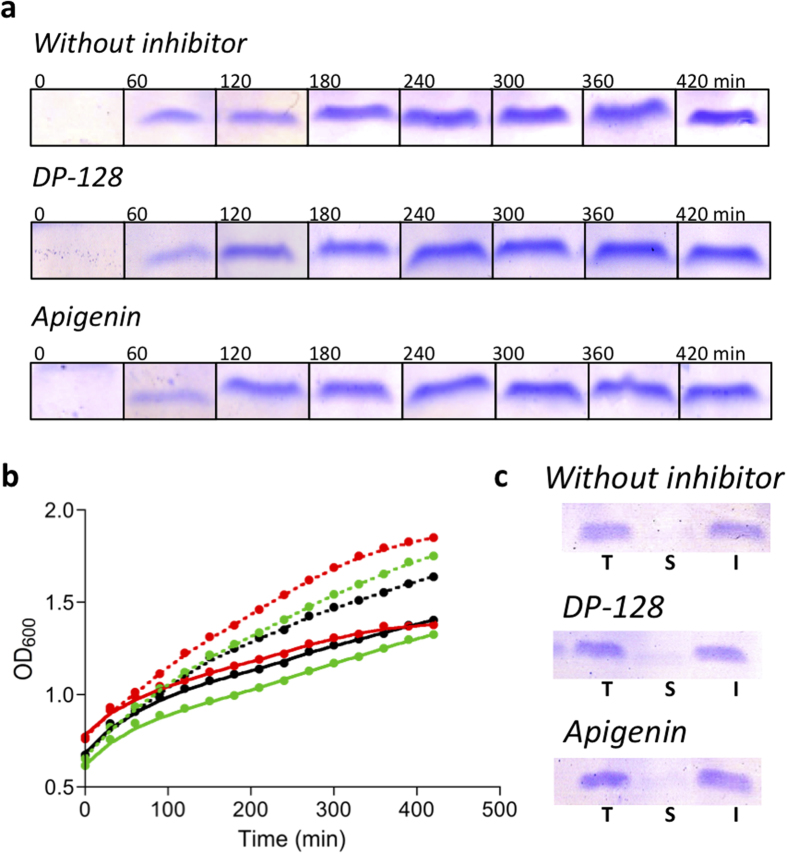
Aβ40 expression and bacterial growth in the absence and presence of inhibitors. (**a**) Protein expression in bacterial cultures along the time tracked by tricine-SDS-PAGE. (**b**) Bacterial growth monitored by optical density at 600 nm (OD_600_); the ODs were measured in triplicate and the standard errors were less than 5%. In black, red and green, in the absence (control) and presence of 10 μM DP-128 and apigenin, respectively. Solid and dashed lines show induced and non-induced cultures, respectively. (**c**) Aβ40 distribution in bacteria: Total (T), soluble (S) and insoluble (I) fraction of Aβ40 at end-point of kinetics.

Aβ40 and Aβ42 are the main components of the senile plaques. However, the physicochemical properties of Aβ40 *e.g.* higher solubility and lower aggregation propensity relative to Aβ42 – have made it the peptide of choice in kinetic *in vitro* assays. Because the over-expression of Aβ42 in bacteria results in the formation of a large amount of oligomeric species^28^, we propose here to use the Aβ40 variant, which does not form oligomers in bacteria^28^, to screen the anti-aggregation effect of two potential inhibitors of the amyloid fibril polymerization. However, Aβ42 aggregation kinetics have been additionally analyzed to assess the effect of the inhibitors in both Aβ variants. We show herein the high efficiency and performance of our ultra rapid *in vivo* screening method for anti-Alzheimer anti-amyloid drugs in bacteria over-expressing Aβ40 and Aβ42 in the presence or absence of two potential inhibitors of Aβ aggregation; DP-128, a chlorotacrine-based inhibitor of Aβ42 and tau aggregation, and apigenin, a natural flavone with demonstrated activity against insulin amyloids (structures in Supplementary Fig. 1)^31^,^32^. In order to show the differences in the Th-S spectra of bacteria over-expressing or not Aβ (*viz.* with or without IBs containing Aβ in amyloid conformation, respectively) as well as the potential tracking of amyloid formation *in vivo*, we measured the kinetics of induced (over-expressing Aβ40) and non-induced (non-overexpressing Aβ40) bacterial cultures in the presence and absence of inhibitors, by tracking the Th-S fluorescence spectra along the time in a range from 460 to 600 nm, exciting at 440 nm. Under these conditions, the formation of Aβ40 and Aβ42 IBs with time leads to unequivocal changes between non-induced and induced cultures as a consequence of the Th-S staining of IBs (Supplementary Fig. 2). In the non-induced samples the maximal fluorescence of Th-S as a consequence of the interaction with bacteria is at ~523 nm (Supplementary Fig. 2a,c,e). However, in the induced samples without inhibitor we observe the progressive appearance of a new peak (non-observable in the non-induced samples) at ~495 nm, arising from the Th-S binding to gradually formed Aβ IBs (Supplementary Fig. 2b); Interestingly, this peak is reduced in the induced samples in the presence of inhibitors as a result of the reduction of Aβ aggregation (Supplementary Fig. 2d,f). When the spectra of non-induced cultures were subtracted from those of Aβ over-expressing cultures along the time-course, we obtained the typical amyloid band displaying a maximal peak at ~485 nm (Fig. 1 and Supplementary Fig. 3). Importantly, we observed a diminution of the amyloid band in the samples with inhibitor (Fig. 1b,c) in comparison with the samples without inhibitor (Fig. 1a); with this fact being indicative of a reduction of the amyloid structures in the bacterial cells that grow in the presence of inhibitors (Supplementary Fig. 3a). The relative fluorescence data at 485 nm can be transformed into amyloid concentration. To quantify the obtained peaks we built a Th-S–Aβ40 standard curve using *in vitro* preformed Aβ40 amyloid fibrils (Supplementary Fig. 4b,c)^33^. Then, the Aβ40 amount in amyloid-like conformation that is present in IBs can be easily and precisely quantified by simple interpolation of the Th-S relative fluorescence data from the time-course reaction at 485 nm in the standard curve (Fig. 2). In order to discard differences in bacterial growth and protein expression level as a consequence of the presence of the inhibitors, we evaluated Aβ40 concentration along the time (by tricine-SDS-PAGE) (Fig. 3a and Supplementary Fig. 5) and bacterial growth (by OD at 600 nm) in the presence and absence of each inhibitor (Fig. 3b). The SDS-PAGE band corresponding to Aβ peptide has been previously identified by western blotting using 6E10 Aβ antibody^28^. As shown in Fig. 3a,b, no significant differences in expression and growth patterns were observed, hence confirming that these inhibitors do not interfere with these parameters. In the case that the potential drug interfered with these parameters the differences would have to be taken into account to normalize further amyloid aggregation kinetics. Complementarily, we checked the Aβ40 distribution between soluble and insoluble fractions (i.e. between soluble and aggregated Aβ40) at the end-point of kinetics. As shown in Fig. 3c, Aβ40 is present almost exclusively in the insoluble fraction. Interestingly, no significant differences in the Aβ40 distribution between soluble and insoluble fractions were observed in the absence and presence of inhibitors (Fig. 3c).

Amyloid kinetics can be mechanistically described as a nucleation dependent polymerization process^34^, wherein initially soluble species are associated forming nuclei in a thermodynamically disfavored stage, with these preformed nuclei acting as templates of soluble species that are favorably added to form first amyloid fibrils and eventually mature fibers^34^,^35^. *In vitro* and *in vivo* systems are being extensively used for exploring and modeling the kinetics of Aβ aggregation, which is one of the most important target for drug discovery and development^13^,^15^,^35^,^36^,^37^,^38^,^39^,^40^,^41^,^42^,^43^,^44^,^45^. Within a wide range of models, this kind of processes can be mathematically addressed as an autocatalytic reaction, considering the nucleation (*k*_n_) and elongation (*k*_e_) constants of the process, and associated kinetic times: lag time of the thermodynamically disfavored phase (*t*_0_), maximal growth (*t*_1/2_) and final time of the amyloid aggregation (*t*_1_)^35^. When the obtained data from the aggregation kinetics are analyzed using the autocatalytic model, the effect of each inhibitor can be easily quantified in kinetic and thermodynamic terms. Thus, *k*_n_ displays reductions of ~1.7 and ~1.5-fold in the presence of DP-128 and apigenin, respectively, leading to elongations of ∼25% in *t*_0_ for both inhibitors. Reductions in *k*_n_ rate denote an inhibitory action in the nucleation phase, i.e. the formation of amyloid nuclei is partially hindered as a consequence of the presence of the inhibitor delaying the appearance of the first amyloid-like species. In contrast, *k*_e_^app^ is increased ∼1.6 and ∼2-fold in the presence of DP-128 and apigenin, respectively, but without significant changes in *t*_1/2_ and *t*_1_. Increments in the *k*_e_^app^ should be interpreted as an increment in the elongation rate, i.e. as an acceleration of the elongation of the fibrils. However, this is only due to the fact that *k*_e_^app^ has been calculated using the final Aβ40 amyloid amount of each aggregation kinetic, it being 6.0, 4.0, and 3.2 μM for the control without inhibitor, DP-128, and aginenin, respectively (Table 1). In brief, at a same elongation rate, the less monomers are available to form fibrils, the sooner the reaction ends. However, as shown in Table 1, when [Aβ40_fibrils_]·*k*_e_ is calculated similar *k*_e_ are obtained (~0.017 min^−1^). In consequence, *t*_1/2_ and *t*_1_ remain essentially unaltered in the presence of inhibitors. In kinetic terms, the obtained data suggest that both inhibitors act by delaying the amyloid aggregation and hindering the nuclei formation. In addition, the final amounts of amyloid-like Aβ40 were reduced in 33 and 46% for DP-128 and apigenin, respectively (Fig. 2 and Table 1). The obtained data show that DP-128 and apigenin act essentially in the nucleation whereas elongation is almost not altered. However, both inhibitors led to a remarkable reduction in the final amount of amyloid aggregates under our experimental conditions. As shown in Supplementary Fig. 6 Th-S staining could be directly assessed by optical fluorescence microscopy. Whereas non-induced cells do not show any Th-S fluorescence (Supplementary Fig. 6a), induced cells in the absence of inhibitor (Supplementary Fig. 6b) display high fluorescence as a consequence of Th-S staining so that the Aβ40 IBs can be easily detected. In the presence of active inhibitors the fluorescence is drastically reduced (Supplementary Fig. 6c,d) denoting a reduction of amyloid amount in IBs. Interestingly, similar inhibitions are obtained when the Aβ42 variant has been tested. Indeed, as shown in Supplementary Fig. 7, DP-128 and apigenin lead to inhibitions of Aβ42 aggregation of 28 and 45%, respectively.

In summary, the proposed method, directly tracking the Th-S relative fluorescence in bacterial cells, allows the *in vivo* monitoring of the activity of potential anti-amyloid drugs in an extremely short time (<24 h), shedding light on the inhibitor behavior and enabling the determination of IC_50_ values if different inhibitor concentrations are used. Interestingly, the present method allows simultaneous acquisition of the kinetic and thermodynamic parameters of each process, enabling the fast classification of inhibitors by accurately quantifying their inhibitory activity in each stage of the amyloid aggregation process. The reported method could be easily adapted to real-time micro-plate readers, thereby enabling high-throughput screenings with the simultaneous tracking of up to 1536 samples. Importantly, the method can be potentially used for the screening of anti-amyloid drugs against all conformational diseases by simply changing the amyloid-prone protein over-expressed in bacteria.

## Methods

### Chemicals and bacterial media

DP-128, *N*-{8-[(6-chloro-1,2,3,4-tetrahydroacridin-9-yl)amino]octyl}-5-(4-chlorophenyl)-1,2,3,4-tetrahydrobenzo[*h*][1,6]naphthyridine-9-carboxamide, was prepared as previously reported^31^. The natural flavone apigenin (4′,5,7-trihydroxyflavone) and all other general chemicals were purchased from Sigma-Aldrich. Compounds for bacterial media were purchased from Pronadisa. M9 minimal medium; For 100 mL: 10 mL salts 10 × (0.68 g Na_2_HPO_4_, 0.30 g KH_2_PO_4_, 0.05 g NaCl, 0.10 g NH_4_Cl), 0.2 mL 1 M MgSO_4_, 0.2 mL 50 mM CaCl_2_, 2.5 mL 20% glucose and 87.1 mL H_2_O.

### Preparation of soluble Aβ40 peptide

Aβ40 (trifluoroacetic acid salt) was obtained from Bachem. A stock solution was prepared by dissolving 1 mg of Aβ40 in 0.5 mL of 1,1,1,3,3,3-hexafluoro-2-propanol (HFIP), which had been dried at 4 °C over molecular sieves Type 4A and then centrifuged (15,000 *g* for 15 min) to remove molecular sieve dust. The solution was incubated under stirring at room temperature for 1 h in sealed vials, bath-sonicated for 30 min, and stirred for an additional hour at room temperature. Then, the solution was kept at 4 °C for 30 min to avoid solvent evaporation when aliquoting. Once the sample was aliquoted, HFIP was removed by evaporation under a gentle stream of nitrogen, leaving a slightly yellow film. The samples of soluble Aβ40 peptide were frozen at −80 °C. Frozen aliquots were re-suspended in 50 μL of anhydrous dimethyl sulfoxide (DMSO) and bath-sonicated for 10 min. Sonication was crucial to remove any traces of un-dissolved seeds that may resist solubilization. Aliquots of Aβ were re-suspended in 950 μL of PBS at pH 7.4. The final buffer contained 5% (v/v) DMSO^46^. The final concentration of Aβ was adjusted by UV absorbance considering a molar absorptivity of 1490 mol^−1^·dm^3^·cm^−1^ at 280 nm using a UV-2401 PC UV-Vis spectrophotometer from Shimadzu.

### *In vitro* Aβ40 aggregation

Aggregation of initially soluble Aβ40 was carried out for 48 h at 37 °C and 1,400 rpm using a Thermomixer from Eppendorf. The amount of insoluble amyloid fibrils at the end-time of the reaction was quantified after centrifugation at 14,000 *g* for 30 min. As previously observed, the insoluble fraction represented 90% of the initial Aβ40, remaining 10% as soluble^33^. This correction was taken into account in the determination of the amyloid stock concentration.

The standard curve was built by sequential dilution of the stock solution. The relative fluorescence of the samples was determined using a final concentration of Th-S of 250 μM and 75, 50, 25, 12.5, 6.25, 3.125, 1.56, 0.8, 0.4 and 0 μM of *in vitro* fibrils of Aβ40 preformed under the same conditions as the kinetics.

### Cloning and expression of Aβ peptide

*Escherichia coli* competent cells BL21 (DE3) were transformed with the pET28a vector from Novagen carrying the DNA sequence of Aβ40 or Aβ42. Because of the addition of the initiation codon ATG in front of gene, the over-expressed peptide contains an additional methionine residue at its N terminus (MDAEFRHDSGYEVHHQKLVFFAEDVGSNKGAIIGLMVGGVV for Aβ40 and MDAEFRHDSGYEVHHQKLVFFAEDVGSNKGAIIGLMVGGVVIA for Aβ42). For overnight culture preparation, 10 mL of M9 minimal medium containing 50 μg·mL^−1^ of kanamycin were inoculated with a single colony of BL21 (DE3) bearing the plasmid to be expressed at 37 °C. For expression of the Aβ peptide, 100 μL of overnight culture (providing a starting OD_600_ of 0.1–0.2) was used to inoculate 9.9 mL of fresh M9 minimal medium containing kanamycin and Th-S for a final concentration of 50 μg·mL^−1^ and 250 μM, respectively, and 10 μL of the inhibitors in 10 mM stock solution DMSO. Note that in the samples without inhibitor 10 μL of DMSO had to be added. Then, bacterial cultures were grown at 37 °C and 250 rpm. When OD_600_ reached ~0.7, 10 μL of isopropyl 1-thio-β-D-galactopyranoside (IPTG) at 1 M were added to induce Aβ over-expression. Then, 200 μL of sample were collected every 30 min during all time-course of the kinetics and fluorescence and absorbance were determined.

As “positive control” we used induced bacterial cells bearing Aβ plasmid (over-expressing Aβ) without inhibitor, displaying the maximal potential aggregation of the Aβ peptide in bacteria. As “negative control” we used non-induced bacterial cells bearing Aβ plasmid without inhibitor, showing the minimal expression of Aβ in the cells. However, to discard potential effects of the inhibitor in the Th-S signal (e.g. when the inhibitor also displays certain fluorescence in Th-S range), a “negative control” of each inhibitor was performed using non-induced bacterial cells bearing Aβ plasmid in the presence of each inhibitor. Importantly, this is highly useful to discard potential effects of the inhibitors in the cell growth (by OD_600_).

### Quantification of Aβ40 in amyloid conformation in bacterial cells

Because non-induced cultures display relative fluorescence in the Th-S emission range (460–600 nm) when exciting at 440 nm (Supplementary Fig. 2a,c,e), these spectra would have to be removed from Th-S fluorescence spectra of induced cultures (Supplementary Fig. 2b,d,f) to quantify the amount of Aβ40 in amyloid-like conformation. Since OD_600_ are known at each kinetic time, induced cultures (over-expressing Aβ40) could be easily corrected by subtracting the spectrum of non-induced cells with similar OD_600_, thus obtaining Th-S amyloid band (Fig. 1).

As shown in Supplementary Fig. 4b,c, the standard curve of *in vitro* Aβ40 fibrils shows a linear relationship between Aβ40 fibril concentration and Th-S relative fluorescence at 485 nm (the maximum of the amyloid band). The interpolation of Th-S fluorescence of *in vivo* kinetics at 485 nm in the standard curve allows the easy transformation from fluorescence into amyloid concentration.

### Aβ40 expression levels in bacterial cells

Aβ40 concentration in cell cultures in the presence and absence of inhibitor was assessed by tricine-SDS-PAGE. 300 μL of induced bacterial cells were collected each hour during all time-course of the kinetics. Then, the samples were concentrated by gentle centrifugation obtaining OD_600_ of 8. 30 μL of concentrated sample were added to 4× loading buffer (250 mM Tris-HCl pH 6.8, 10% SDS, 0.008% Bromophenol Blue, 40% glycerol and 2.86 M β-mercaptoethanol). Finally, the samples were placed at 95 °C for 10 min. Denatured samples were analyzed using 16% tricine SDS-PAGE (Fig. 3a and Supplementary Fig. 5).

In order to check the Aβ40 aggregation in bacteria, soluble and insoluble protein fractions were required. Then, 1 mL of concentrated sample from the end-point of each kinetic was pelleted by centrifugation at 14,000 rpm (20 min, 4 °C). The pellet containing the cells was frozen at −80 °C for at least 2 h. Then, cell pellets were re-suspended in 1 mL of phosphate buffer saline 1× (PBS) and sonicated using a SONICS Vibra Cell^TM^ from Vibra-Cell-Sonics & Materials, Inc. at 30% during 1 min (with intervals of 1 seg sonication/0.5 seg stand-by). Soluble and insoluble fractions were separated by centrifugation at 14,000 rpm (20 min, 4 °C). The insoluble fraction was re-suspended in 1 mL PBS 1 ×. Finally, the samples were analyzed by tricine-SDS-PAGE (Fig. 3c).

### Thioflavin-S (Th-S) Steady-State Fluorescence

Relative fluorescence of Th-S binding to Aβ was checked using an Aminco Bowman Series 2 luminescence spectrophotometer from Aminco-Bowman with an excitation wavelength of 440 nm and emission range from 460 to 600 nm. The emission at 485 nm was recorded for further determination of the amyloid-like concentration. Excitation and emission slit widths of 4 nm were used and spectra were acquired at 1 nm intervals and 500 nm·min^−1^ rates and 875 V^47^. Importantly, in order to compare the obtained data, the voltage must always be known and not changed.

### Aggregation assay analysis

The amyloid aggregation in bacteria may be studied as an autocatalytic reaction using the equation:




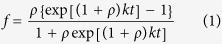




under the boundary condition of *t* = 0 and *f* = 0, where *k* = *k*_e_*a* (when *a* is the protein concentration) and * ρ* represents the dimensionless value to describe the ratio of *k*_n_ to *k.* By non-linear regression of *f* against *t*, values of *ρ* and *k* are obtained, and from them the rate constants, *k*_e_ (elongation constant) and *k*_n_ (nucleation constant)^35^. Note that because the concentration of Aβ is not constant in the bacterial cells during the protein over-expression, we have arbitrarily considered as *a* the Aβ concentration of the final time-course, obtaining apparent values for *k*_e_, which allow quantitative comparison of kinetics. The extrapolation of the linear portion of the sigmoid curve to abscissa (*f* = 0), and to the highest ordinate value of the fitted plot, afforded two values of time (*t*_0_ and *t*_1_), which correspond to the lag time and to the end-time reaction. The time at which half of the protein was aggregated (i.e., when *f* = 0.5) was considered the time of half-aggregation (*t*_1/2_)^35^.

### Optical fluorescence microscopy

Bacterial cells overexpressing Aβ40 were incubated 1 h with 125 μM of Th-S. Th-S was removed by centrifugation and the cells were re-suspended in PBS twice and placed on a microscope slide. Th-S fluorescence was detected using a Leitz DMIRB microscope under UV light using a GFP filter (excitation filter BP480/40 and emission filter BP527/30)^48^.

## Additional Information

**How to cite this article**: Espargaró, A. *et al*. Ultra rapid *in vivo* screening for anti-Alzheimer anti-amyloid drugs. *Sci. Rep.* **6**, 23349; doi: 10.1038/srep23349 (2016).

## Supplementary Material

Supplementary Information

## Figures and Tables

**Table 1:** Kinetic and thermodynamic parameters of Aβ40 amyloid aggregation.

**Inhibitor**	**Without**	**DP-128** a	**Apigenin** a
*k*_n_ (10^6^·min^−1^)	200.5	118.5	135.0
*k*_e_^app^ (M^−1^·min^−1^)^b^,^c^	2723.5	4427.8	5336.5
[Aβ40_fibrils_]·*k*_e_ (min^−1^)	0.0164	0.0179	0.0173
*t*_0_ (min)	112.0	140.6	144.3
*t*_1/2_ (min)	268.3	279.4	279.4
*t*_1_ (min)	424.5	418.3	414.6
[Aβ40_fibrils_] (μM)^d^	6.0	4.0	3.2

^a^Inhibition parameters at 10 μM of inhibitor.

^b^Since Aβ40 concentration is not constant along the aggregation process, the *k*_e_ are apparent.

^c^In order to calculate the *k*_e_^app^, the final Aβ40 amyloid amount of each aggregation kinetic has been considered.

^d^Aβ40 concentration in amyloid conformation at end-point of the time-course.

## References

[b1] Chiti F. & Dobson C. M. Protein misfolding, functional amyloid, and human disease. Annu Rev Biochem 75, 333–366 (2006). 16756495 10.1146/annurev.biochem.75.101304.123901

[b2] Reitz C., Brayne C. & Mayeux R. Epidemiology of Alzheimer disease. Nat Rev Neurol 7, 137–152, doi: 10.1038/nrneurol.2011.2 (2011). 21304480 PMC3339565

[b3] Prince M., Wimo A., Guerchet M., Ali G.-C., Wu Y.-T. & Prina M. World Alzheimer Report 2015. The global impact of dementia. An analysis of prevalence, incidence, cost & trends; Alzheimer’s Disease International: London, http://www.alz.co.uk, (2015) (Accessed:15/02/2016)

[b4] Huang Y. & Mucke L. Alzheimer mechanisms and therapeutic strategies. Cell 148, 1204–1222, doi: 10.1016/j.cell.2012.02.040 (2012). 22424230 PMC3319071

[b5] Ballard C. . Alzheimer’s disease. Lancet 377, 1019–1031, doi: 10.1016/S0140-6736(10)61349-9 (2011). 21371747

[b6] Skaper S. D. Alzheimer’s disease and amyloid: culprit or coincidence? Int Rev Neurobiol 102, 277–316, doi: 10.1016/B978-0-12-386986-9.00011-9 (2012). 22748834

[b7] Bucciantini M. . Inherent toxicity of aggregates implies a common mechanism for protein misfolding diseases. Nature 416, 507–511, doi: 10.1038/416507a (2002). 11932737

[b8] Godyn J., Jonczyk J., Panek D. & Malawska B. Therapeutic strategies for Alzheimer’s disease in clinical trials. Pharmacol Rep 68, 127–138, doi: 10.1016/j.pharep.2015.07.006 (2016). 26721364

[b9] Jia Q., Deng Y. & Qing H. Potential therapeutic strategies for Alzheimer’s disease targeting or beyond β-amyloid: insights from clinical trials. Biomed Res Int 2014, 837157, doi: 10.1155/2014/837157 (2014). 25136630 PMC4124758

[b10] Aisen P. S. The development of anti-amyloid therapy for Alzheimer’s disease : from secretase modulators to polymerisation inhibitors. CNS Drugs 19, 989–996, doi: 19122 (2005). 16332141 10.2165/00023210-200519120-00002

[b11] Lannfelt L. . Perspectives on future Alzheimer therapies: amyloid-β protofibrils-a new target for immunotherapy with BAN2401 in Alzheimer’s disease. Alzheimers Res Ther 6, 16, doi: 10.1186/alzrt246 (2014). 25031633 PMC4054967

[b12] Meyer-Luehmann M. . Rapid appearance and local toxicity of amyloid-β plaques in a mouse model of Alzheimer’s disease. Nature 451, 720–724, doi: 10.1038/nature06616 (2008). 18256671 PMC3264491

[b13] Tokuraku K., Marquardt M. & Ikezu T. Real-time imaging and quantification of amyloid-β peptide aggregates by novel quantum-dot nanoprobes. PLoS One 4, e8492, doi: 10.1371/journal.pone.0008492 (2009). 20041162 PMC2794548

[b14] Burgold S., Filser S., Dorostkar M. M., Schmidt B. & Herms J. *In vivo* imaging reveals sigmoidal growth kinetic of β-amyloid plaques. Acta Neuropathol Commun 2, 30, doi: 10.1186/2051-5960-2-30 (2014). 24678659 PMC4050984

[b15] Meisl G. . Differences in nucleation behavior underlie the contrasting aggregation kinetics of the Aβ40 and Aβ42 peptides. Proc Natl Acad Sci USA 111, 9384–9389, doi: 10.1073/pnas.1401564111 (2014). 24938782 PMC4084462

[b16] Ullah G., Demuro A., Parker I. & Pearson J. E. Analyzing and Modeling the Kinetics of Amyloid Β Pores Associated with Alzheimer’s Disease Pathology. PLoS One 10, e0137357, doi: 10.1371/journal.pone.0137357 (2015). 26348728 PMC4562663

[b17] Villar-Pique A., Espargaro A., Ventura S. & Sabate R. *In vivo* amyloid aggregation kinetics tracked by time-lapse confocal microscopy in real-time. Biotechnol J, doi: 10.1002/biot.201500252 (2015). 26580000

[b18] Xu X. . Prevention of β-amyloid induced toxicity in human iPS cell-derived neurons by inhibition of Cyclin-dependent kinases and associated cell cycle events. Stem Cell Res 10, 213–227, doi: 10.1016/j.scr.2012.11.005 (2013). 23305945

[b19] Hou X. Q. . A novel assay for high-throughput screening of anti-Alzheimer’s disease drugs to determine their efficacy by real-time monitoring of changes in PC12 cell proliferation. Int J Mol Med 33, 543–549, doi: 10.3892/ijmm.2013.1608 (2014). 24378397 PMC3926499

[b20] Bartolini M. . Insight into the kinetic of amyloid β (1–42) peptide self-aggregation: elucidation of inhibitors’ mechanism of action. ChemBioChem 8, 2152–2161, doi: 10.1002/cbic.200700427 (2007). 17939148

[b21] Bartolini M. . Kinetic characterization of amyloid-β 1–42 aggregation with a multimethodological approach. Anal Biochem 414, 215–225, doi: 10.1016/j.ab.2011.03.020 (2011). 21435333

[b22] Haass C. & Selkoe D. J. Soluble protein oligomers in neurodegeneration: lessons from the Alzheimer’s amyloid β-peptide. Nat Rev Mol Cell Biol 8, 101–112, doi: nrm2101 (2007). 17245412 10.1038/nrm2101

[b23] Prangkio P., Yusko E. C., Sept D., Yang J. & Mayer M. Multivariate analyses of amyloid-β oligomer populations indicate a connection between pore formation and cytotoxicity. PLoS One 7, e47261, doi: 10.1371/journal.pone.0047261 (2012). 23077580 PMC3471831

[b24] Bernstein S. L. . Amyloid-β protein oligomerization and the importance of tetramers and dodecamers in the aetiology of Alzheimer’s disease. Nat Chem 1, 326–331, doi: 10.1038/nchem.247 (2009). 20703363 PMC2918915

[b25] Ventura S. & Villaverde A. Protein quality in bacterial inclusion bodies. Trends Biotechnol 24, 179–185, doi: S0167-7799(06)00052-7 (2006). 16503059 10.1016/j.tibtech.2006.02.007

[b26] Wang L., Maji S. K., Sawaya M. R., Eisenberg D. & Riek R. Bacterial inclusion bodies contain amyloid-like structure. PLoS Biol 6, e195, doi: 10.1371/journal.pbio.0060195 (2008). 18684013 PMC2494559

[b27] de Groot N. S., Sabate R. & Ventura S. Amyloids in bacterial inclusion bodies. Trends Biochem Sci 34, 408–416 (2009). 19647433 10.1016/j.tibs.2009.03.009

[b28] Dasari M. . Bacterial inclusion bodies of Alzheimer’s disease β-amyloid peptides can be employed to study native-like aggregation intermediate states. ChemBioChem 12, 407–423, doi: 10.1002/cbic.201000602 (2011). 21290543

[b29] Pouplana S. . Thioflavin-S staining of bacterial inclusion bodies for the fast, simple, and inexpensive screening of amyloid aggregation inhibitors. Curr Med Chem 21, 1152–1159, doi: CMC-EPUB-56032 (2014). 24059241 10.2174/09298673113206660256

[b30] Espargaro A., Sabate R. & Ventura S. Thioflavin-S staining coupled to flow cytometry. A screening tool to detect *in vivo* protein aggregation. Mol Biosyst 8, 2839–2844, doi: 10.1039/c2mb25214g (2012). 22868714

[b31] Di Pietro O. . Tetrahydrobenzo[*h*][1,6]naphthyridine-6-chlorotacrine hybrids as a new family of anti-Alzheimer agents targeting β-amyloid, tau, and cholinesterase pathologies. Eur J Med Chem 84, 107–117, doi: 10.1016/j.ejmech.2014.07.021 (2014). 25016233

[b32] Amini R., Yazdanparast R. & Bahramikia S. Apigenin reduces human insulin fibrillation *in vitro* and protects SK-N-MC cells against insulin amyloids. Int J Biol Macromol 60, 334–340, doi: 10.1016/j.ijbiomac.2013.06.013 (2013). 23777711

[b33] Sabate R. & Estelrich J. Pinacyanol as effective probe of fibrillar β-amyloid peptide: comparative study with Congo Red. Biopolymers 72, 455–463, doi: 10.1002/bip.10485 (2003). 14587068

[b34] Jarrett J. T. & Lansbury P. T. Jr. Seeding “one-dimensional crystallization” of amyloid: a pathogenic mechanism in Alzheimer’s disease and scrapie? Cell 73, 1055–1058 (1993). 8513491 10.1016/0092-8674(93)90635-4

[b35] Sabate R., Gallardo M. & Estelrich J. An autocatalytic reaction as a model for the kinetics of the aggregation of β-amyloid. Biopolymers 71, 190–195, doi: 10.1002/bip.10441 (2003). 12767118

[b36] Cohen S. I., Vendruscolo M., Dobson C. M. & Knowles T. P. Nucleated polymerization with secondary pathways. II. Determination of self-consistent solutions to growth processes described by non-linear master equations. J Chem Phys 135, 065106, doi: 10.1063/1.3608917 (2011). 21842955 PMC5036541

[b37] Cohen S. I. . Nucleated polymerization with secondary pathways. I. Time evolution of the principal moments. J Chem Phys 135, 065105, doi: 10.1063/1.3608916 (2011). 21842954 PMC5017532

[b38] Lomakin A., Chung D. S., Benedek G. B., Kirschner D. A. & Teplow D. B. On the nucleation and growth of amyloid β-protein fibrils: detection of nuclei and quantitation of rate constants. Proc Natl Acad Sci USA 93, 1125–1129 (1996). 8577726 10.1073/pnas.93.3.1125 PMC40042

[b39] Lomakin A., Teplow D. B., Kirschner D. A. & Benedek G. B. Kinetic theory of fibrillogenesis of amyloid β-protein. Proc Natl Acad Sci USA 94, 7942–7947 (1997). 9223292 10.1073/pnas.94.15.7942 PMC21534

[b40] Pallitto M. M. & Murphy R. M. A mathematical model of the kinetics of β-amyloid fibril growth from the denatured state. Biophys J 81, 1805–1822, doi: S0006-3495(01)75831-6 (2001). 11509390 10.1016/S0006-3495(01)75831-6 PMC1301655

[b41] Murphy R. M. & Pallitto M. M. Probing the kinetics of β-amyloid self-association. J Struct Biol 130, 109–122, doi: 10.1006/jsbi.2000.4253 (2000). 10940219

[b42] Murphy R. M. Kinetics of amyloid formation and membrane interaction with amyloidogenic proteins. Biochim Biophys Acta 1768, 1923–1934, doi: S0005-2736(06)00491-3 (2007). 17292851 10.1016/j.bbamem.2006.12.014

[b43] Knowles T. P. . An analytical solution to the kinetics of breakable filament assembly. Science 326, 1533–1537, doi: 10.1126/science.1178250 (2009). 20007899

[b44] Ferrone F. Analysis of protein aggregation kinetics. Methods Enzymol 309, 256–274 (1999). 10507029 10.1016/s0076-6879(99)09019-9

[b45] Cohen S. I., Vendruscolo M., Dobson C. M. & Knowles T. P. Nucleated polymerization with secondary pathways. III. Equilibrium behavior and oligomer populations. J Chem Phys 135, 065107, doi: 10.1063/1.3608918 (2011). 21842956 PMC5017531

[b46] Sabate R. & Estelrich J. Evidence of the existence of micelles in the fibrillogenesis of β-amyloid peptide. J Phys Chem B 109, 11027–11032 (2005). 16852343 10.1021/jp050716m

[b47] Sola I. . Novel Levetiracetam Derivatives That Are Effective against the Alzheimer-like Phenotype in Mice: Synthesis, *in Vitro, ex Vivo*, and *in Vivo* Efficacy Studies. J Med Chem 58, 6018–6032, doi: 10.1021/acs.jmedchem.5b00624 (2015). 26181606

[b48] Perez-Areales F. J. . Shogaol-huprine hybrids: dual antioxidant and anticholinesterase agents with β-amyloid and tau anti-aggregating properties. Bioorg Med Chem 22, 5298–5307, doi: 10.1016/j.bmc.2014.07.053 (2014). 25156301

