# Supplementary material for: Ultra rapid in vivo screening for anti-Alzheimer anti-amyloid drugs
Source: Sci Rep. 2016 Mar 22;6:23349. doi: 10.1038/srep23349 (PMC4802339; doi:10.1038/srep23349)
Supplement: Supplementary Information [file srep23349-s1.pdf]

Ultra rapid *in vivo* screening for anti-Alzheimer anti-amyloid drugs

Alba Espargaró[1], Aina Medina[1], Ornella Di Pietro[2], Diego Muñoz-Torrero[2] & Raimon Sabate[1,*]

[1]Department of Physical Chemistry, School of Pharmacy, and Institute of Nanoscience and Nanotechnology (IN[2]UB), University of Barcelona, Spain.
[2]Laboratory of Pharmaceutical Chemistry (CSIC Associated Unit), School of Pharmacy, and Institute of Biomedicine (IBUB), University of Barcelona, Spain.
*rsabate@ub.edu

Additional information

Supplementary Figure 1. Chemical structures of DP-128 and apigenin.

Supplementary Figure 2. Th-S relative fluorescence along the time-course amyloid kinetics. (a, b) In the absence of inhibitor, **(c, d)** In the presence of 10 µM DP-128 and **(e, f)** in the presence of 10 µM apigenin. Left panel and right panels show non-induced and induced bacterial cultures, respectively. In green and red, the initial (0 min) and final (480 min) time-course, respectively. Th-S relative fluorescence measurements were performed in triplicate and the standard errors were less than 5%.

Supplementary Figure 3. Th-S fluorescence spectra of bacterial cells overexpressing Aβ42. In blue, induced bacterial cells (over-expressing Aβ40); in green, non-induced bacterial cells (non-expressing recombinant Aβ40); in red, amyloid band obtained by subtraction of non-induced cells spectrum in induced cells one.

Supplementary Figure 4. Aβ40 standard curve and amyloid amount quantification: From relative fluorescence to amyloid concentration. (a) Th-S relative fluorescence enhancement along the time-course kinetics. In black, red and green, in the absence (control) and presence of 10 µM DP-128 and apigenin, respectively. **(b)** Th-S relative fluorescence of *in vitro* Aβ40 fibrils **(c)** Standard curve representing Th-S relative fluorescence *versus* Aβ40 aggregated fibrils. Th-S relative fluorescence measurements were performed in triplicate and the standard errors were less than 5%.

Supplementary Figure 5. Aβ40 expression. Protein expression in bacterial cultures along the time tracked by tricine-SDS-PAGE.

Supplementary Figure 6. Optical fluorescence microscopy. Images under UV light of bacterial cells overexpressing Aβ40 peptide stained with Th-S, in the absence and in the presence of anti-aggregating compounds. Scale bar corresponds to 5 *µ*m.

Supplementary Figure 7. Aβ42 amyloid concentration along the time-course kinetics and amyloid concentration at end-point of the time-course. In black, red and green, in the absence (control) and presence of 10 µM DP-128 and apigenin, respectively. The Aβ40 concentrations were measured in triplicate and the standard errors were less than 5%.

DP-128

N-{8-[(6-chloro-1,2,3,4-tetrahydroacridin-9-yl)amino]octyl}-5-(4-chlorophenyl)-1,2,3,4-tetrahydrobenzo[*h*][1,6]naphthyridine-9-carboxamide

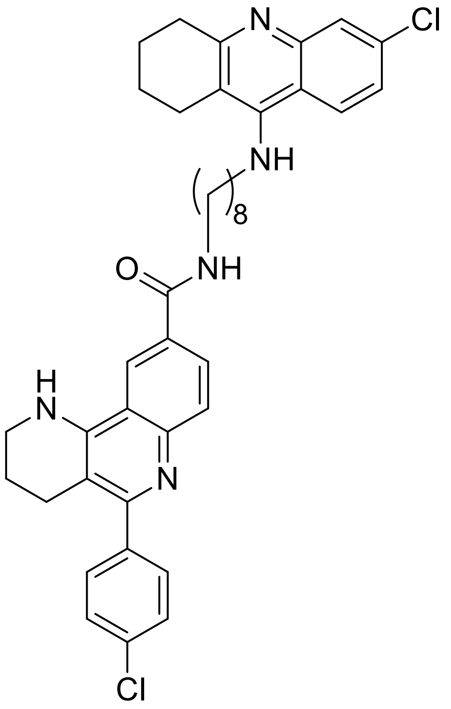

Apigenin

5,7-Dihydroxy-2-(4-hydroxyphenyl)-4-benzopyrone

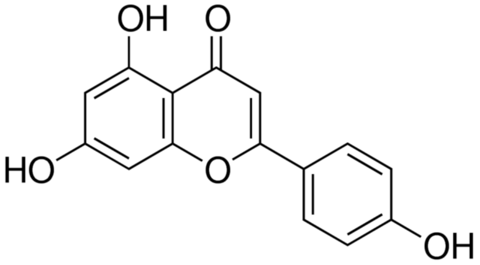

Supplementary Figure 1. **Chemical structures of DP-128 and apigenin.**

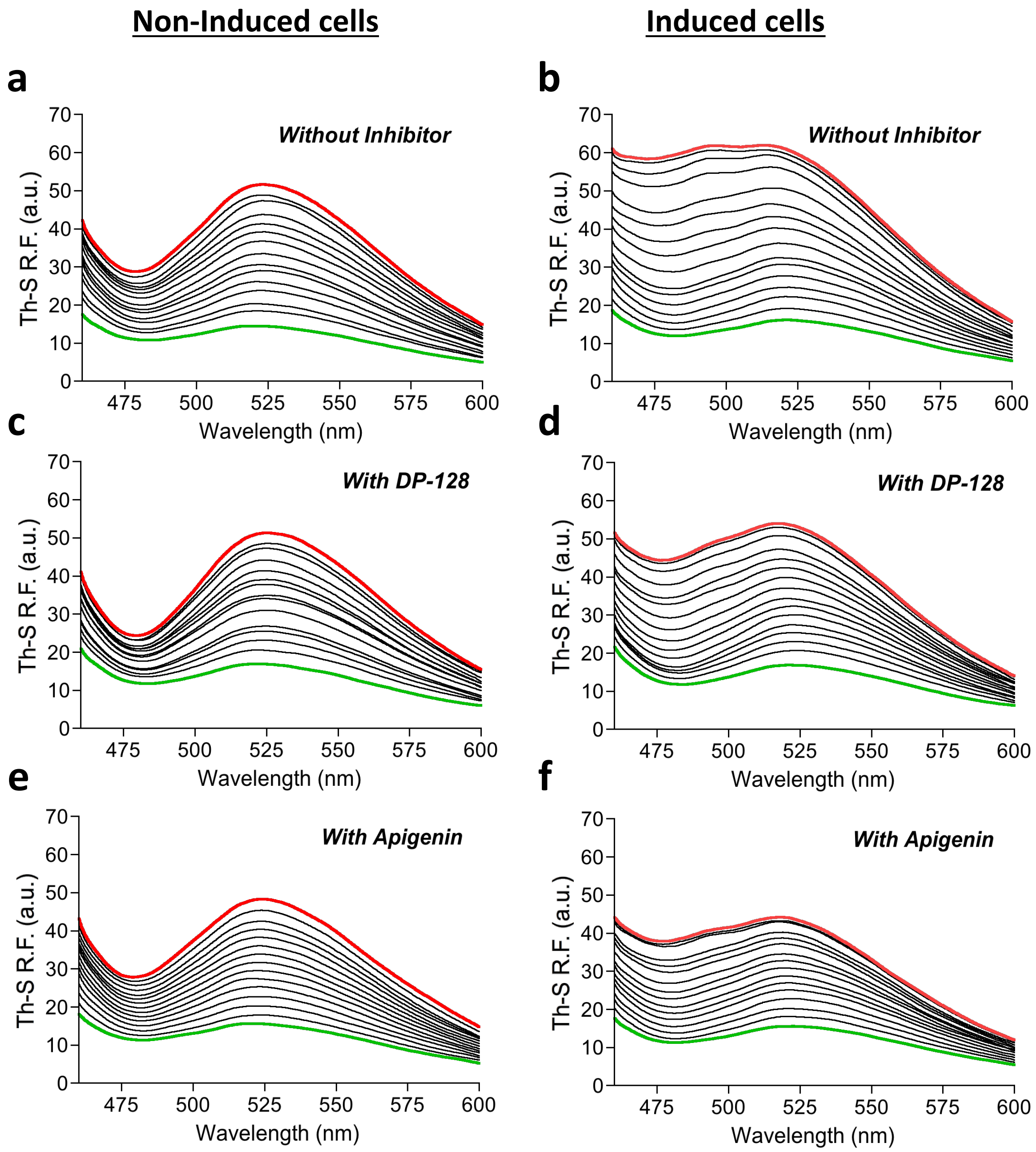

Supplementary Figure 2. **Th-S relative fluorescence along the time-course amyloid kinetics. (a, b)** In the absence of inhibitor, **(c, d)** In the presence of 10 μM DP-128 and **(e, f)** in the presence of 10 μM apigenin. Left panel and right panels show non-induced and induced bacterial cultures, respectively. In green and red, the initial (0 min) and final (480 min) time-course, respectively. Th-S relative fluorescence measurements were performed in triplicate and the standard errors were less than 5%.

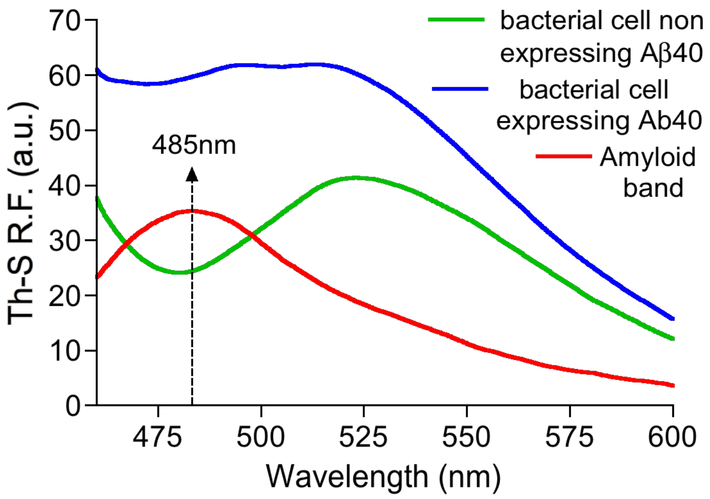

Supplementary Figure 3. Th-S fluorescence spectra of bacterial cells overexpressing Aβ42. In blue, induced bacterial cells (over-expressing Aβ40); in green, non-induced bacterial cells (non-expressing recombinant Aβ40); in red, amyloid band obtained by subtraction of non-induced cells spectrum in induced cells one.

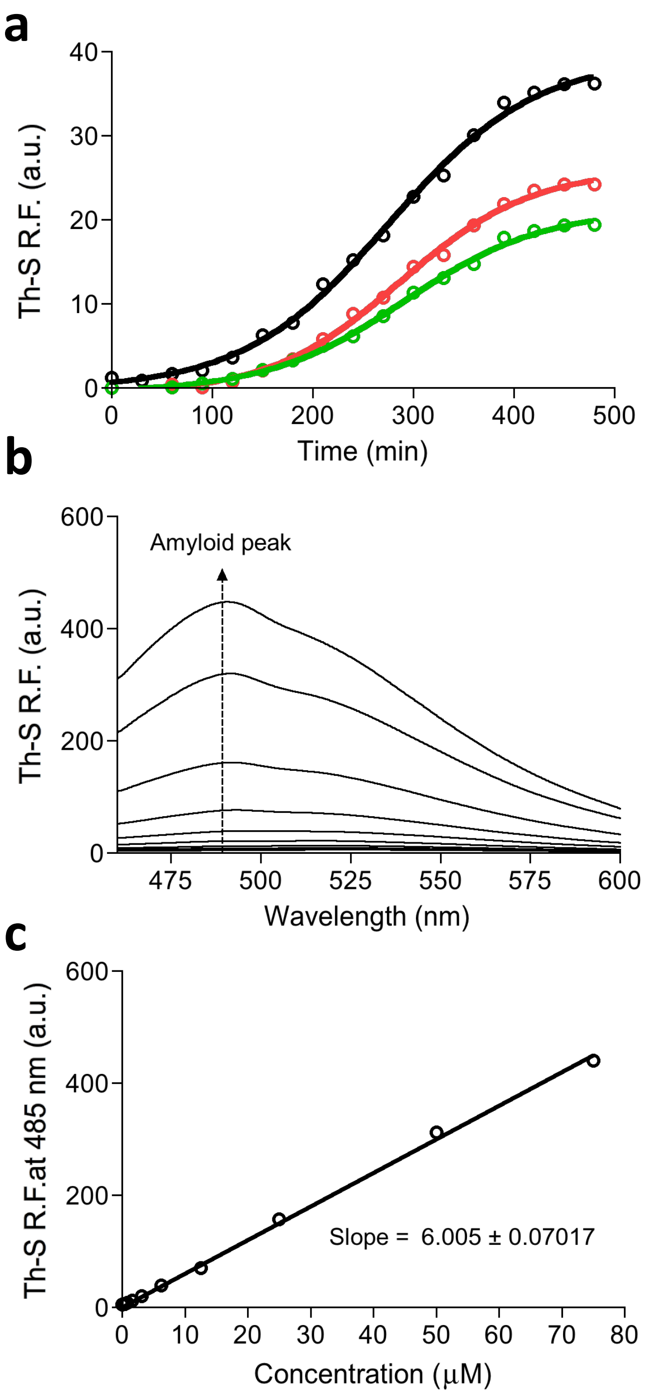

Supplementary Figure 4. Aβ40 standard curve and amyloid amount quantification: From relative fluorescence to amyloid concentration. (a) Th-S relative fluorescence enhancement along the time-course kinetics. In black, red and green, in the absence (control) and presence of 10 μM DP-128 and apigenin, respectively. **(b)** Th-S relative fluorescence of *in vitro* Aβ40 fibrils **(c)** Standard curve representing Th-S relative fluorescence *versus* Aβ40 aggregated fibrils. Th-S relative fluorescence measurements were performed in triplicate and the standard errors were less than 5%.

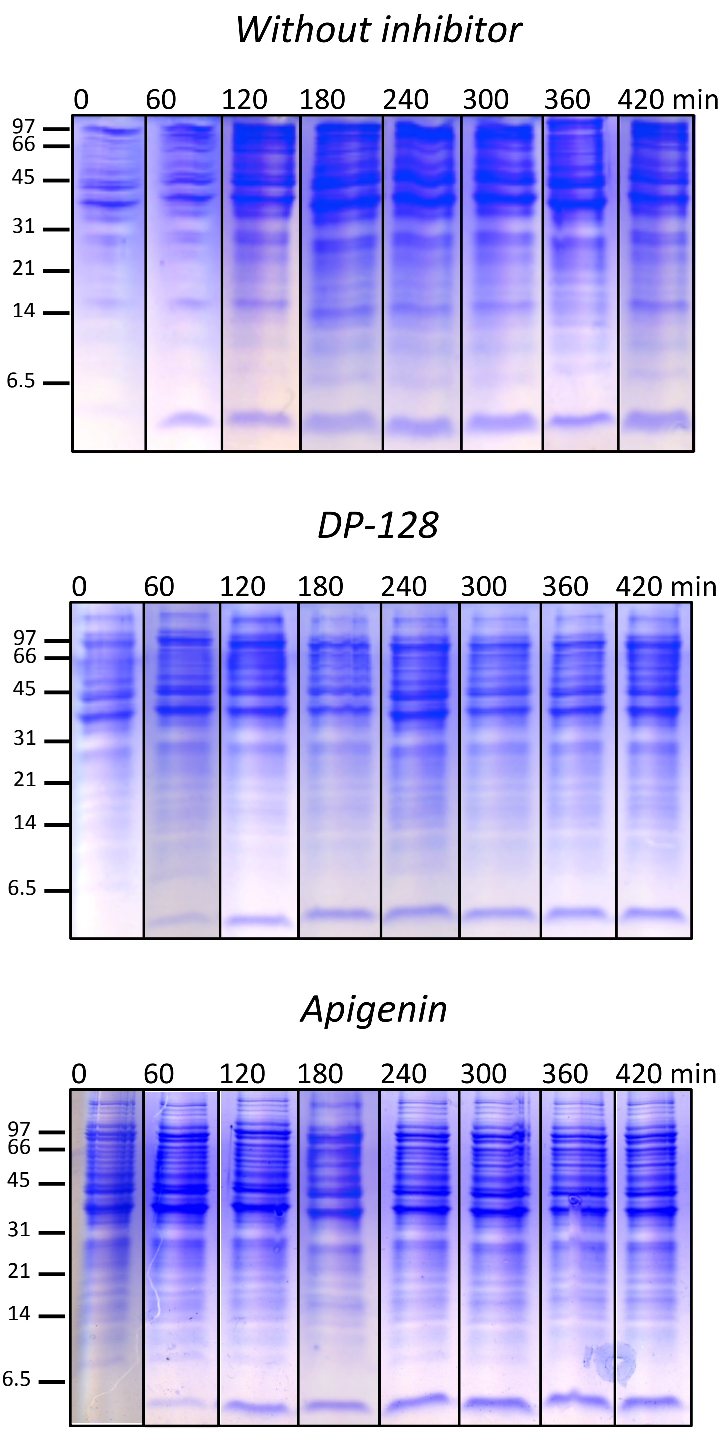

Supplementary Figure 5. **Aβ40 expression.** Protein expression in bacterial cultures along the time tracked by tricine-SDS-PAGE.

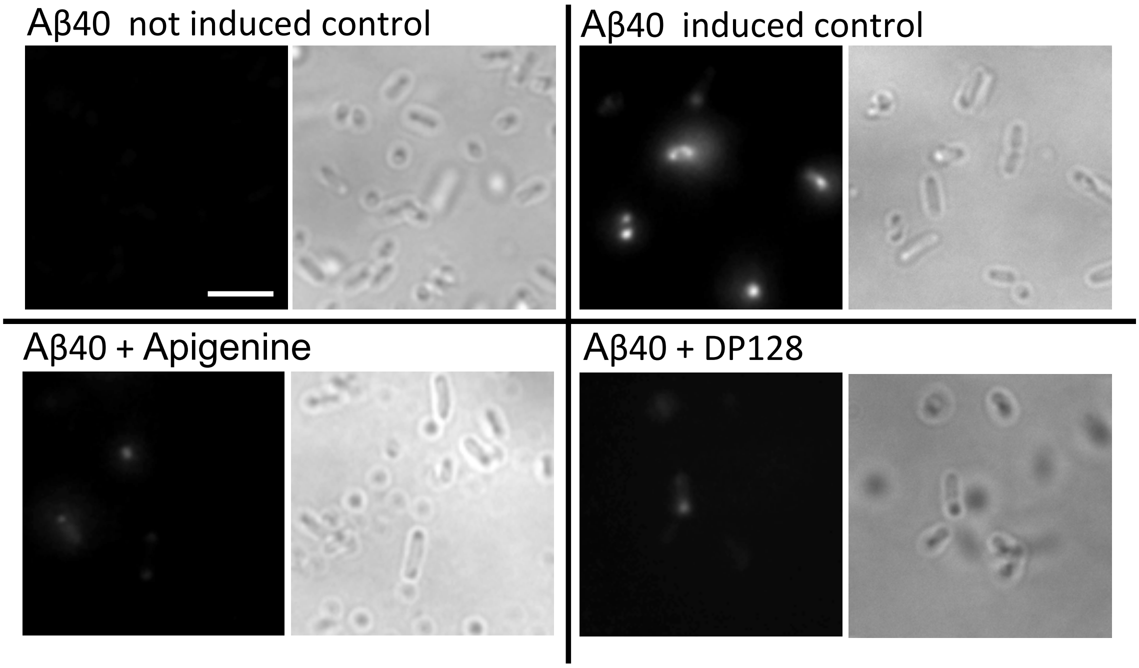

Supplementary Figure 6. **Optical fluorescence microscopy**. Images under UV light of bacterial cells overexpressing Aβ40 peptide stained with Th-S, in the absence and in the presence of anti-aggregating compounds. Scale bar corresponds to 5 μm.

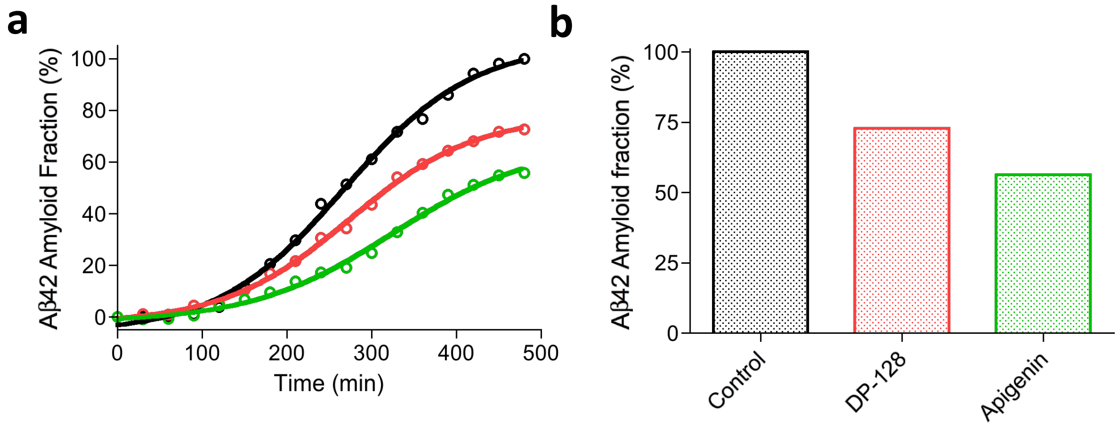

Supplementary Figure 7. **Aβ42 amyloid concentration along the time-course kinetics and amyloid concentration at end-point of the time-course.** In black, red and green, in the absence (control) and presence of 10 μM DP-128 and apigenin, respectively. The Aβ40 concentrations were measured in triplicate and the standard errors were less than 5%.
